# Supplementary material for: Heptanoate Improves Compensatory Mechanism of Glucose Homeostasis in Mitochondrial Long-Chain Fatty Acid Oxidation Defect
Source: Nutrients. 2023 Nov 5;15(21):4689. doi: 10.3390/nu15214689 (PMC10649308; doi:10.3390/nu15214689)
Supplement: Supplementary file 1 [file nutrients-15-04689-s001.zip › nutrients-2652511-supplementary.pdf]

Supplementary data

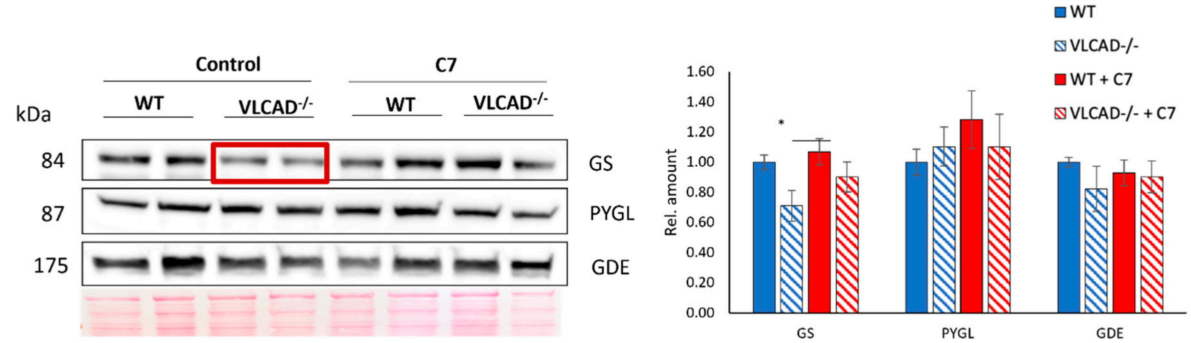

Figure S1. VLCAD$^{-/-}$ mice affected protein expression of glycogen metabolism. *n* = 10–12. Mice groups consisted of both sexes. Significant differences were measured using two-way ANOVA test; * adjusted *p* value (*q*) < 0.05

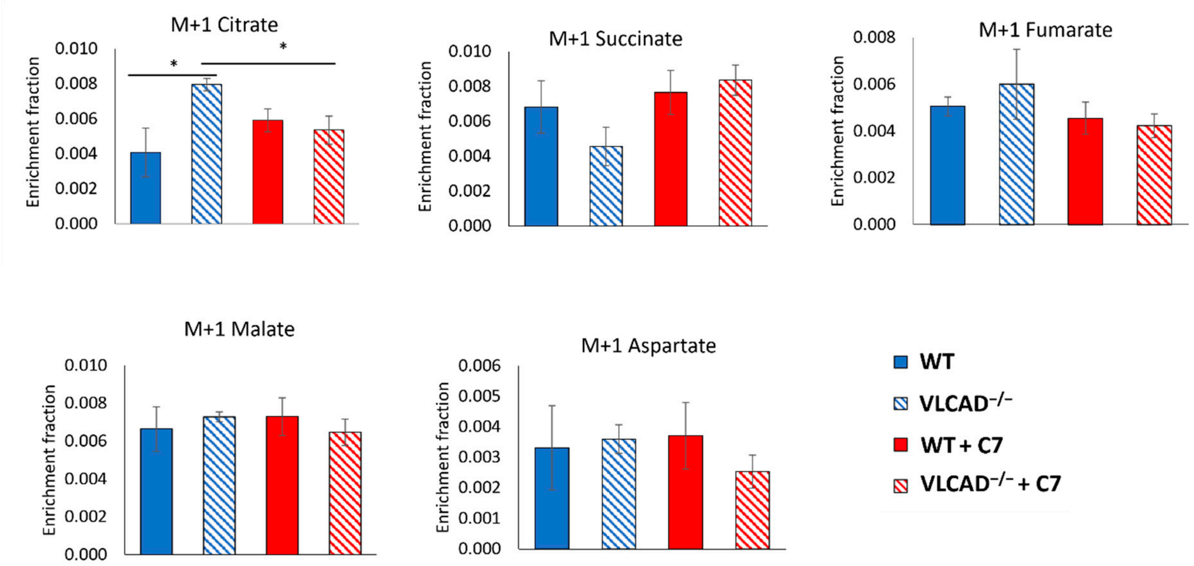

Figure S2. The fractional of M+1 enrichment in liver TCA cycle intermediates. *n* = 10–12. Mice groups consisted of both sexes. Significant differences were measured using two-way ANOVA test; * adjusted *p* value (*q*) < 0.05.

Table S1. The fractional ^{13}C enrichment in the example metabolite (pyruvate) of unlabeled sample (A) and labeled sample (B) after isotope correction.

A

	**average**				**s.e.m.**			
	WT	VLCAD$^{-/-}$	WT+C7	VLCAD$^{-/-}$+C7	WT	VLCAD$^{-/-}$	WT + C7	VLCAD$^{-/-}$ + C7
M+0	1.000	1.000	1.000	1.000	0.000	0.000	0.001	0.001
M+1	0.000	0.000	0.000	0.000	0.000	0.000	0.001	0.001
M+2	0.000	0.000	0.000	0.000	0.000	0.000	0.000	0.001
M+3	0.000	0.000	0.000	0.000	0.000	0.000	0.000	0.000

B

	**average**				**s.e.m.**			
	WT	VLCAD$^{-/-}$	WT+C7	VLCAD$^{-/-}$+C7	WT	VLCAD$^{-/-}$	WT+C7	VLCAD$^{-/-}$+C7
M+0	0.996	0.995	0.996	0.996	0.000	0.001	0.001	0.000
M+1	0.002	0.001	0.001	0.002	0.000	0.001	0.000	0.000
M+2	0.001	0.001	0.001	0.001	0.000	0.000	0.000	0.000
M+3	0.002	0.004	0.003	0.002	0.000	0.000	0.001	0.000

Table S2. The fractional ^{13}C enrichment in TCA cycle metabolite: citrate (A), succinate (B), malate (C), and fumarate (D).

A

	**average**				**s.e.m.**			
	WT	KO	C7_WT	C7_KO	WT	KO	C7_WT	C7_KO
M+0	0.993	0.988	0.992	0.992	0.001	0.001	0.001	0.001
M+1	0.004	0.008	0.006	0.005	0.001	0.000	0.001	0.001
M+2	0.004	0.004	0.002	0.003	0.000	0.001	0.001	0.001
M+3	0.000	0.001	0.001	0.001	0.000	0.000	0.000	0.000
M+4	0.000	0.000	0.000	0.000	0.000	0.000	0.000	0.000
M+5	0.000	0.000	0.000	0.000	0.000	0.000	0.000	0.000

B

	**average**				**s.e.m.**			
	WT	KO	C7_WT	C7_KO	WT	KO	C7_WT	C7_KO
M+0	0.989	0.988	0.988	0.987	0.002	0.001	0.002	0.001
M+1	0.007	0.005	0.008	0.008	0.001	0.001	0.001	0.001
M+2	0.003	0.007	0.004	0.006	0.001	0.001	0.001	0.001
M+3	0.000	0.001	0.000	-0.001	0.000	0.000	0.000	0.000
M+4	0.001	0.000	0.000	0.000	0.000	0.000	0.000	0.000

C

	**average**				**s.e.m.**			
	WT	KO	C7_WT	C7_KO	WT	KO	C7_WT	C7_KO

M+0	0.990	0.988	0.991	0.990	0.001	0.001	0.001	0.001
M+1	0.005	0.006	0.005	0.004	0.000	0.001	0.001	0.001
M+2	0.004	0.005	0.004	0.005	0.001	0.000	0.001	0.001
M+3	0.000	0.001	0.001	0.001	0.000	0.000	0.000	0.000
M+4	0.000	0.000	0.000	0.000	0.000	0.000	0.000	0.000

D

	**average**				**s.e.m.**			
	WT	KO	C7_WT	C7_KO	WT	KO	C7_WT	C7_KO
M+0	0.992	0.989	0.990	0.992	0.002	0.001	0.002	0.001
M+1	0.007	0.007	0.007	0.006	0.001	0.000	0.001	0.001
M+2	0.002	0.004	0.002	0.001	0.001	0.001	0.001	0.001
M+3	0.000	0.000	0.000	0.001	0.000	0.000	0.000	0.000
M+4	0.000	0.000	0.000	0.000	0.000	0.000	0.000	0.000
